# Transcription factor 7-like 2 (TCF7L2) variant is associated with familial breast cancer risk: a case-control study

**DOI:** 10.1186/1471-2407-6-268

**Published:** 2006-11-17

**Authors:** Barbara Burwinkel, Kalai S Shanmugam, Kari Hemminki, Alfons Meindl, Rita K Schmutzler, Christian Sutter, Barbara Wappenschmidt, Marion Kiechle, Claus R Bartram, Bernd Frank

**Affiliations:** 1 Helmholtz-University Group Molecular Epidemiology, German Cancer Research Center, DKFZ, Heidelberg, Germany; 2 Division of Molecular Genetic Epidemiology, German Cancer Research Center, DKFZ, Heidelberg, Germany; 3 Center for Family Medicine, Karolinska Institute, Huddinge, Sweden; 4 Department of Gynaecology and Obstetrics, Klinikum rechts der Isar at the Technical University, Munich, Germany; 5 Division of Molecular Gynaeco-Oncology, Department of Gynaecology and Obstetrics, Clinical Center University of Cologne, Germany; 6 Center of Molecular Medicine Cologne (CMMC), University Hospital of Cologne, Germany; 7 Institute of Human Genetics, University of Heidelberg, Heidelberg, Germany

## Abstract

**Background:**

The transcription factor 7-like 2 (TCF7L2) is a critical component of the Wnt/β-catenin pathway. Aberrant *TCF7L2 *expression modifies Wnt signaling and mediates oncogenic effects through the upregulation of *c-MYC *and *cyclin D*. Genetic alterations in *TCF7L2 *may therefore affect cancer risk. Recently, *TCF7L2 *variants, including the microsatellite marker DG10S478 and the nearly perfectly linked SNP rs12233372, were identified to associate with type 2 diabetes.

**Methods:**

We investigated the effect of the *TCF7L2 *rs12255372 variant on familial breast cancer (BC) risk by means of TaqMan allelic discrimination, analyzing *BRCA1/2 *mutation-negative index patients of 592 German BC families and 735 control individuals.

**Results:**

The T allele of rs12255372 showed an association with borderline significance (OR = 1.19, 95% C.I. = 1.01-1.42, *P *= 0.04), and the Cochran-Armitage test for trend revealed an allele dose-dependent association of rs12255372 with BC risk (*P*_trend _= 0.04).

**Conclusion:**

Our results suggest a possible influence of *TCF7L2 *rs12255372 on the risk of familial BC.

## Background

The transcription factor 7-like 2 (*TCF7L2 *alias *TCF-4*) gene product, a HMG box transcription factor, is part of the Wnt/β-catenin signaling cascade, one of the key mechanisms of development and growth regulation in the cell [1]. Grant *et al*. [2] have identified *TCF7L2 *as a novel candidate gene for type 2 diabetes and reported an association of the microsatellite marker DG10S478 within intron 3. The associated tetranucleotid repeat was shown to have six alleles, namely alleles 0, 4, 8, 12, 16 and 20. The combined non-zero alleles of DG10S478 (referred to as X) were associated with an increased risk in three independent populations (Danish, Icelandic and US). The composite at-risk allele X was nearly perfectly correlated with the T allele of the single nucleotide polymorphism (SNP) rs12255372, while the G allele was linked to allele 0. Consequently, the rs12255372 T allele showed association with type 2 diabetes as well [2]. Recent studies have confirmed these findings, revealing strong association between rs12255372 and type 2 diabetes in Dutch and US cohorts [3,4].

There is accumulating evidence that aberrant activation of the Wnt/β-catenin pathway has oncogenic effects [5,6]. Wnt signaling results in increased cytosolic levels of β-catenin which is released from the APC/Axin/GSK3β degradation complex and translocated into the nucleus to bind TCF7L2 [1,2,6]. The transcriptionally competent β-catenin/TCF7L2 complexes provoke an excessive expression of TCF7L2 target genes, such as the *cyclin D *and *c-MYC *oncogenes, which is a common feature in human cancers, including breast cancer (BC). This supports the biological significance and clinical relevance of the Wnt/β-catenin signaling pathway in carcinogenesis [7-9].

Given these facts, we investigated the effect of the rs12255372 polymorphism on familial BC risk.

## Methods

### Study population

We analyzed *TCF7L2 *rs12255372 in 592 German familial BC cases and 735 control individuals. The BC cases comprised unrelated female index patients (19 to 87 years of age, median age 45) without mutations in the high-penetrance genes *BRCA1 *and *BRCA2*. Mutations in the open reading frame of *BRCA1 *and *BRCA2 *were excluded by applying denaturing high performance liquid chromatography (DHPLC) on all exons, followed by direct sequencing of conspicuous exons. Cases were collected during the years 1996–2005 through the Institute of Human Genetics (Heidelberg, Germany), the Department of Gynaecology and Obstetrics (Cologne, Germany) and the Department of Medical Genetics (Munich, Germany). According to the German Consortium for Hereditary Breast and Ovarian Cancer, BC cases were divided into six categories based on family history: (A1) families with two or more breast cancer cases including at least two cases with onset below the age of 50 years (39.0%); (A2) families with at least one male breast cancer case (0.7%); (B) families with at least one breast cancer and one ovarian cancer case (18.1%); (C) families with at least two breast cancer cases including one case diagnosed before the age of 50 years (25.5%); (D) families with at least two breast cancer cases diagnosed after the age of 50 years (8.4%) and (E) single cases of breast cancer diagnosed before the age of 35 years (8.3%) [10,11]. The *TCF7L2 *rs12255372 analysis comprised one index case per family. DNA of further family members to evaluate segregation of the variant with BC risk was not available.

The corresponding control series consisted of healthy, unrelated and ethnically matched blood donors (26 to 68 years of age, median age 49) sharing the ethnic background with the patients. They were recruited in 2004 and 2005 by the Institute of Transfusion Medicine and Immunology (Mannheim, Germany). The study was approved by the Ethics Committee of the University of Heidelberg (Heidelberg, Germany), and written informed consent was obtained from all individuals.

### SNP selection

Grant *et al*. investigated five SNPs located within a 92.1 kb linkage disequilibrium (LD) block, encompassing parts of introns 3 and 4 and the whole of exon 4 [2]. We selected rs12255372 which was the most highly correlated SNP to the associated microsatellite marker DG10S478 (r^2 ^= 0.95) for genotyping.

### Detection of TCF7L2 rs12255372 genotypes

Genotyping of *TCF7L2 *rs12255372 was performed by TaqMan allelic discrimination as described before [12]. Primers and probes were provided by the assay-by-design service (Applied Biosystems, Foster City, CA). The corresponding sequences are available upon request. Genotyping errors were excluded by re-genotyping ≥ 10% of the samples with a concordance rate of 100%.

### Statistical analysis

Genotype-specific odds ratios (OR), 95% confidence intervals (95% C.I.) and *P *values were computed by unconditional logistic regression using the Statistical Analysis System software (Version 9.1.; SAS Institute Inc., Cary, NC). Hardy-Weinberg equilibrium test was undertaken using Pearson's goodness-of-fit chi-square test with one degree of freedom. Power calculation was carried out with the power and sample size software PS [13]. With the present sample size, we had a power of 80% at a significance level of 0.05 to detect an OR of ≥ 1.38.

## Results and discussion

The *TCF7L2 *rs12255372 T allele frequency of the controls (German Caucasians) was in accordance with those published in previous studies (Danish, Dutch, Icelandic and US Caucasians) [2-4], and the frequencies of rs12255372 genotypes were consistent with Hardy-Weinberg equilibrium (*P *= 0.64, Table 1). The minor T allele of rs12255372 was significantly overrepresented in cases, and we found an allelic association with an increased familial BC risk (OR = 1.19, 95% C.I. = 1.01-1.42, *P *= 0.04, Table 1). Given the borderline significance, a finding by chance cannot be excluded. However, according to the Cochran-Armitage test for trend the association was allele dose-dependent (*P*_trend _= 0.04, Table 1), adding consistency to our data.

The strengths of the present study on BC risk are based on a large sample size and a homogeneous study cohort of a single ethnic group. Only *BRCA1*/*2 *mutation-negative familial BC cases were included in order to avoid effects caused by these high-penetrance susceptibility genes. Our study comprised individuals selected for familial BC, since the power of an association study based on cases with a family history of the disease is considerably higher compared to a study using unselected cases [14,15].

This is the first study to investigate *TCF7L2 *as a candidate gene for cancer susceptibility, suggesting a prominent role of *TCF7L2 *variants in human cancers, especially in BC. According to Duval *et al*. [16], mutant *TCF7L2 *stimulates its transcriptional activity synergistically with *APC*/*β-catenin *gene alterations. Moreover, it is predicted to show a reduced binding of the C-terminal binding protein (CtBP) which hence loses its capability to repress TCF7L2 activity [17]. Both hypotheses involve an increase of *TCF7L2 *transcriptional activation, leading to uncontrolled target gene expression. Along the lines of Grant *et al*. [2] who have ruled out exonic mutations, we assume that the linked repeat polymorphism DG10S478 is causative itself or that DG10S478 and rs12255372 are in strong LD with a functional variant affecting transcription, splicing or message stability.

In summary, our data suggest that *TCF7L2 *variants may contribute to the risk of familial BC. Regarding the borderline significance level of our results, confirmation in an independent BC cohort is essential. Moreover, it would be of interest to estimate their impact on further types of human cancer.

## Conclusion

Our data suggest a possible influence of the *TCF7L2 *rs12255372 variant on the risk of familial BC.

## Abbreviations

BC – breast cancer

CtBP – C-terminal binding protein

DHPLC – denaturing high performance liquid chromatography

95% C.I. – 95% confidence interval

OR – odds ratio

SNP – single nucleotide polymorphism

TCF7L2 – transcription factor 7-like 2

TCF-4 – HMG box transcription factor 4

## Competing interests

The author(s) declare that they have no competing interests.

## Authors' contributions

BB designed and coordinated the study and reviewed the manuscript. KSS participated in SNP genotyping. KH participated in the study coordination and revised the manuscript. AM, RKS, CS, BW, MK and CRB collected DNA samples and were responsible for the *BRCA1/2 *mutation screening. BF conducted the experiments, performed data acquisition and interpretation, and drafted the manuscript. All authors read and approved the final version of the submitted manuscript.

## Pre-publication history

The pre-publication history for this paper can be accessed here:



## Figures and Tables

**Table 1:** Genotype and allele frequencies of rs12255372 in unrelated female German *BRCA1/2 *mutation-negative familial breast cancer (BC) patients and healthy, unrelated female control subjects

**Genotype**	**BC Case Patients N (%)**	**Control Subjects N (%)**	**OR [95% C.I.], *P *value**^**a**^
GG	297 (50.2)	408 (55.5)	**1.00**
GT	244 (41.2)	276 (37.6)	1.21 [0.97, 1.53], 0.09
TT	51 (8.6)	51 (6.9)	1.37 [0.91, 2.08], 0.13
GT+TT	295 (49.8)	327 (44.5)	**1.24 [1.00, 1.54], 0.05**
**Allele**			

G	0.71	0.74	**1.00**
T	0.29	0.26	**1.19 [1.01, 1.42], 0.04**

**Cochran-Armitage trend test**	***P*_trend _= 0.04**		

^a^Odds ratios (OR) with 95% confidence intervals (95% C.I.) and respective *P *values were computed by unconditional logistic regression using the Statistical Analysis System software (SAS version 9.1.; SAS Institute Inc., Cary, NC). Adjustment for age did not change the ORs, assuming that the distribution of the *TCF7L2 *rs12255372 genotypes is age-independent.
